# A Phenotyping Method of Giant Cells from Root-Knot Nematode Feeding Sites by Confocal Microscopy Highlights a Role for *CHITINASE-LIKE 1* in *Arabidopsis*

**DOI:** 10.3390/ijms19020429

**Published:** 2018-01-31

**Authors:** Javier Cabrera, Rocio Olmo, Virginia Ruiz-Ferrer, Isidro Abreu, Christian Hermans, Isabel Martinez-Argudo, Carmen Fenoll, Carolina Escobar

**Affiliations:** 1 Facultad de Ciencias Ambientales y Bioquímica, Universidad de Castilla-La Mancha, Área de Fisiología Vegetal, Avda, Carlos III, s/n, 45071 Toledo, Spain; javier.cabrerachaves@uclm.es (J.C.); rocio.olmo@uclm.es (R.O.); Virginia.Ruiz@uclm.es (V.R.-F.); carmen.fenoll@uclm.es (C.F.); 2 Centro de Biotecnología y Genómica de Plantas (UPM-INIA), Pozuelo de Alarcón, 28223 Madrid, Spain; isidro.abreu@upm.es; 3 Laboratory of Plant Physiology and Molecular Genetics, Université libre de Bruxelles, Campus Plaine CP 242, Bd du Triomphe, 1050 Brussels, Belgium; Christian.Hermans@ulb.ac.be; 4 Facultad de Ciencias Ambientales y Bioquímica, Universidad de Castilla-La Mancha, Área de Genética, Avda, Carlos III, s/n, 45071 Toledo, Spain; Isabel.MArgudo@uclm.es

**Keywords:** BABB, clearing, confocal microscopy, *CTL1*, giant cells, *Meloidogyne* spp., nodules, syncytia

## Abstract

Most effective nematicides for the control of root-knot nematodes are banned, which demands a better understanding of the plant-nematode interaction. Understanding how gene expression in the nematode-feeding sites relates to morphological features may assist a better characterization of the interaction. However, nematode-induced galls resulting from cell-proliferation and hypertrophy hinders such observation, which would require tissue sectioning or clearing. We demonstrate that a method based on the green auto-fluorescence produced by glutaraldehyde and the tissue-clearing properties of benzyl-alcohol/benzyl-benzoate preserves the structure of the nematode-feeding sites and the plant-nematode interface with unprecedented resolution quality. This allowed us to obtain detailed measurements of the giant cells’ area in an *Arabidopsis* line overexpressing *CHITINASE-LIKE-1* (*CTL1*) from optical sections by confocal microscopy, assigning a role for *CTL1* and adding essential data to the scarce information of the role of gene repression in giant cells. Furthermore, subcellular structures and features of the nematodes body and tissues from thick organs formed after different biotic interactions, i.e., galls, syncytia, and nodules, were clearly distinguished without embedding or sectioning in different plant species (*Arabidopsis*, cucumber or *Medicago*). The combination of this method with molecular studies will be valuable for a better understanding of the plant-biotic interactions.

## 1. Introduction

Plant parasitic nematodes are a major threat to agriculture, due to the ban of effective control agents. In Europe, for instance, Directive 2009/128/EC is banishing chemical nematicides due to their toxicity to the environment and human health (for example in Europe, Directive 2009/128/EC). Sedentary endoparasitic nematodes, like root-knot nematodes (RKNs; *Meloidogyne* spp.) and cyst nematodes (CNs; *Heterodera* spp.; *Globodera* spp.), establish permanent feeding sites within the roots of infected plants [1,2]. More precisely, the RKNs migrate intercellularly from the root elongation zone until they select yet unknown vascular cells that eventually differentiate into feeding cells. These are called giant cells (GCs) as they become more than a hundred times larger than the surrounding cells [3,4,5]. As GCs develop, neighbouring cells proliferate in the vascular cylinder and cortex cells hypertrophy forming a root-knot structure commonly called gall. The RKNs become sedentary within the gall and feed on the GCs until life cycle completion [4,5].

During the last two decades, several approaches have been undertaken to elucidate the origin, development, and functioning of the GCs in direct interaction with the nematodes in model plants, such as *Arabidopsis thaliana* or *Solanum lycopersicum*. Among others aspects, the molecular and cellular level of their cell wall [6], cell cycle regulation [7], metabolism [8,9], or the functional role of crucial transcription factors related to different developmental pathways in GCs [10] have been studied. These studies led to the discovery of several genes involved in the infection process and/or the GCs’ size control. However, tools to assess how plant gene expression relates directly to the GCs’ development are still needed. In this respect, different methods for phenotyping GCs and subcellular structures were developed by using light or confocal microscopy. Semithin sections of galls embedded in resins or waxes are commonly used. In this respect, the combination of semithin sections with image analysis software allowed the three-dimensional reconstruction and volume measurements of GCs induced by *M. javanica* in *Arabidopsis* [3]. Fester et al. [11] used glutaraldehyde-induced auto-fluorescence combined with chloral hydrate clearing for the observation of the GCs and xylem vessels from galls. Additionally, different clearing techniques for the observation of whole gall structures allowed the reconstruction of GCs’ nuclei based on propidium iodide, 4′,6-diamidino-2-phenylindole (DAPI), or SYTOX orange staining [12,13,14]. By using the Transparent plant Organ MEthod for Imaging (TOMEI), based on 2,2′-thiodiethanol (TDE), as a clearing reagent, a strong positive correlation between DNA amount and cell volume, based on data derived from fluorescent proteins and dyes, was observed through confocal microscopy [15]. However, the outlines and content of the GCs were difficult to track in the images, probably due to modest resolution of the images due to the gall thickness. 

Glutaraldehyde is the most effective agent among the aldehydes to generate thermally- and chemically-stable protein crosslinking by reacting with amine groups [16]. It is also a reliable fixative agent for nucleic acids, such as DNA [16]. This crosslink yields a strong green auto-fluorescence (λex/λem 475 nm/525 nm) in cells in neutral solution [17], which has prevented its utilization as a fixative for fluorescent microscopy. Here, we take advantage of the elevated auto-fluorescence derived from the fixation with glutaraldehyde and of the powerful clearing reagent benzyl alcohol and benzyl benzoate (BABB) [18] for a fast, sharp-focused, and in-depth observation by confocal microscopy of the GCs within the galls developed by *M. javanica* in *Arabidopsis* roots at any stage of development. We used this method recently for a general comparison of the GCs’ morphology formed in leaves and roots of *Arabidopsis* [19]. Here we prove that this method allows a high definition visualization of the GCs-nematode interface rarely shown before in entire non-sectioned galls. Furthermore, the neat optical sections obtained were used to quantify the GCs’ size for the study of the role of the *CHITINASE-LIKE 1* (*CTL1*) gene, a GCs repressed gene during the *Arabidopsis*-RKN interaction that has been described as a chitinase-related protein with an impact on root architecture in response to different abiotic conditions [20,21]. Gene repression is a characteristic of early developing GCs observed in several plant species, such as *Arabidopsis* and tomato [22,23]. Accordingly, we have also described the induction of sRNAs in nematode feeding sites at early stages [24], a mechanism proposed to regulate gene repression [25,26,27]. The study of this gene adds useful data to the still scarce information of the role of gene silencing in nematode feeding sites. Moreover, we show that the same fixation-clearing method could be used to directly visualize the nematode inside the root tissues, even at the female stage, as well as the eggs inside the egg masses and inside a nematode–female resistance structure, a cyst, without any dissection step. The method can be extended to the observation of galls of other plant species with thicker root tissues than *Arabidopsis*, such as cucumber. Furthermore, other structures developed by legumes when interacting with symbiotic rhizobia, nodules, and their internal tissues are also clearly distinguished without previous embedding or hand sectioning. 

## 2. Results and Discussion

In recent years, attempts to broaden the knowledge of the interaction between plants and parasitic nematodes have been undertaken. In order to dissect the plant-nematode interaction, one of the main strategies was to study those genes differentially-expressed in the GCs and galls by using holistic approaches [22,23,28,29,30,31,32,33,34]. However, the plant-nematode interaction research faces the challenge to correlate those changes in the gene expression, to variations in the morphological features of nematode-feeding cells (GCs) in those mutant or transgenic plants with altered infection indices. These observations could mean a great advance for the assignation of functional roles to particular genes in GCs. Hence, we used a method that allows for sharp visualization and imaging for 3D reconstruction of GCs to observe the morphology of the plant-nematode interface and subcellular structures critical for GCs’ characterization, such as their nuclei. In addition, morphology of dense reproductive structures, such as *Meloidogyne* spp. females, egg masses, and cysts from *Heterodera* spp., were also imaged from the intact biological material without hand sectioning. Furthermore, we measured the size of GCs from a transgenic line (^OE^CTL1) compared to its control Col-0 by combining a method developed to measure the GCs’ volume from semithin sections of embedded galls in combination with the 3D reconstruction plugin TrakEM2 [3], but based on optical sections from confocal microscopy. This greatly simplified the tedious process of the embedding and sectioning of the galls. 

### 2.1. Galls and GCs Induced by M. javanica in Arabidopsis and Cucumber Are Neatly Observed under Confocal Microscopy at Any Stage of Development

**Figure 1:**
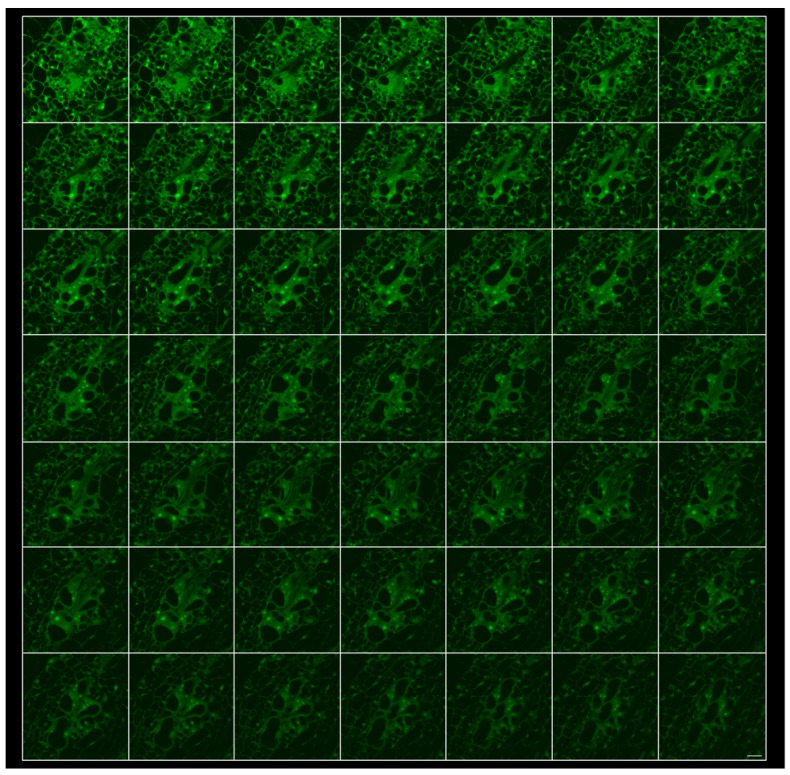
The fixation and clearing method allows the complete sectioning of a gall induced by *Meloidogyne javanica* in *Arabidopsis thaliana* roots at 48–72 h post infection; montage of 49 1-μm optical sections. The images were taken from entire galls after fixation with glutaraldehyde and clearing with BABB. Scale bar: 20 μm. Confocal parameters: PMT 1 (Offs.): −5.70. PMT 1 (HV): 418.22. Excitation beam splitter FW RSP 500. λex/λem 480 nm/500–600 nm. Pinhole (Airy) 1.00.

Hand-dissected galls induced by *M. javanica* in *Arabidopsis* roots were fixed with 3% glutaraldehyde overnight, cleared with BABB as described in the Materials and Methods, section and observed under confocal microscopy (λex/λem: 480 nm/500–600 nm; [19]). A gall at 48–72 h post infection (hpi) could be easily examined entirely obtaining as many optical sections as desired (Figure 1; Video S1). The intact nematode in the centre of the root, the GCs and vascular cells proliferation around the nematode head are clearly distinguishable (Figure 1; 49 sections of 1 μm thickness are shown in Video S1).

**Figure 2:**
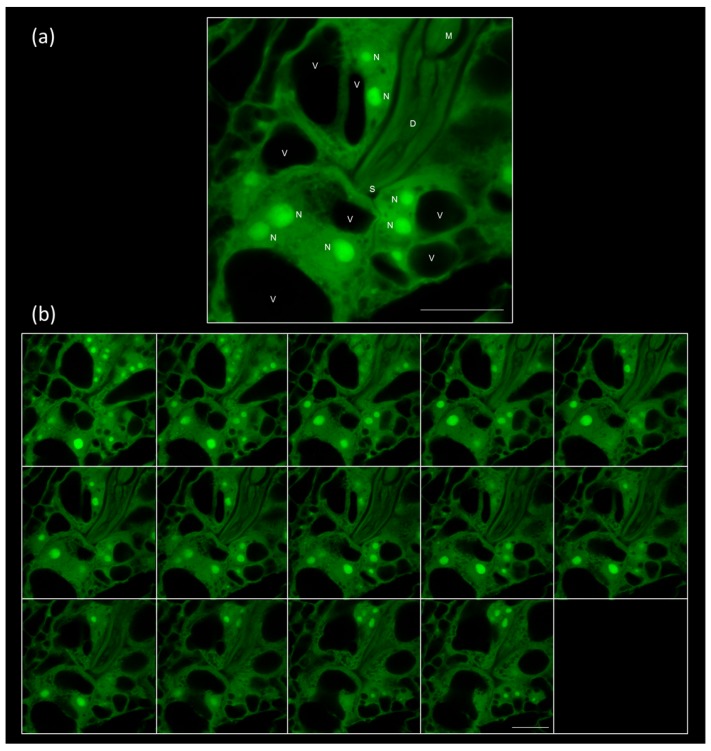
The fixation and clearing method allows the neat visualization of the plant-nematode interface. (**a**) Image of a feeding site at 48–72 h post infection derived from the Z-Stack (Sum) of images 5–9 from (**b**) a montage of 14 1-μm optical sections from a gall induced by *Meloidogyne javanica* in *Arabidopsis thaliana* roots at 48–72 h post infection. The images were taken from entire galls after fixation with glutaraldehyde and clearing with BABB. Scale bars: 20 μm. Confocal parameters: PMT 1 (Offs.): −5.70. PMT 1 (HV): 494.13. Excitation beam splitter FW RSP 500. λex/λem 480 nm/500–600 nm. Pinhole (airy) 1.00. D: dorsal gland; M: metocorpus; N: nucleus; S: stylet; V: vaculole.

At a higher magnification, the nematode head and the protruding stylet puncturing the early developing GCs can be observed (Figure 2a; Video S2). Accordingly, the internal nematode structures, such as the pump chamber, the metacorpus, and the dorsal gland ampulla [35], are also visible (Video S2; Figure 2a). Additionally, multiple and enlarged nuclei, which are characteristic of the GCs [36], and dense cytoplasm, were evident as early as 48–72 hpi (Figure 2a,b; Video S2). 

**Figure 3:**
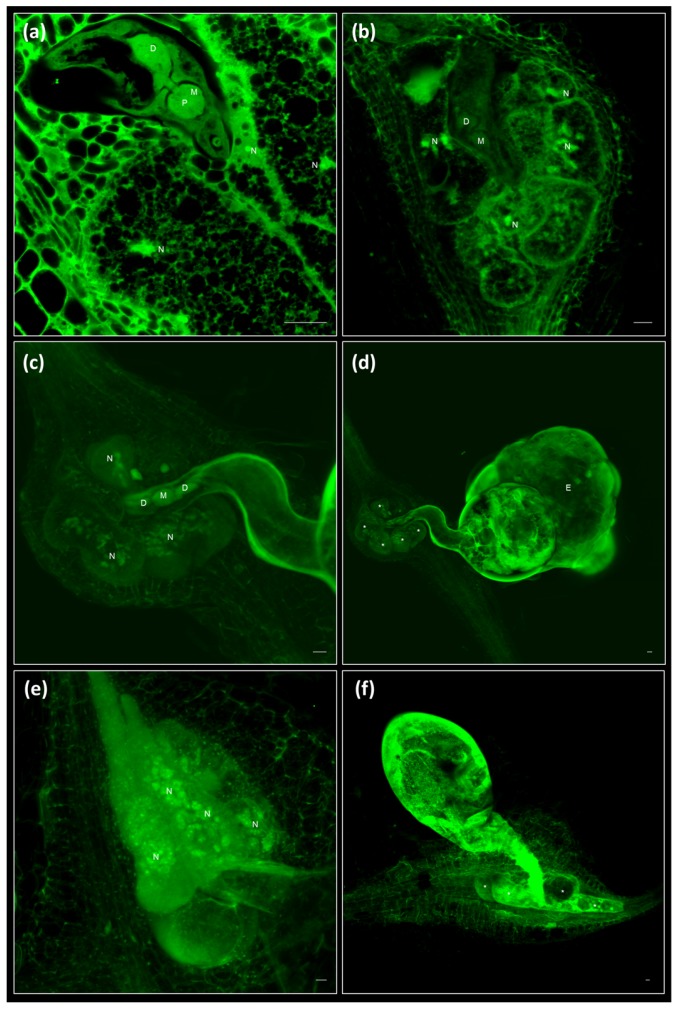
Galls and giant cells, are clearly observed at any stage of development. (**a**) Capture of a gall induced by *Meloidogyne javanica* in *Arabidopsis thaliana* roots at 14 dpi completely sectioned in Video S3. (**b**) Image derived from the Z-Stack (Sum) of 97 images from a gall at 14 dpi (**c**,**d**). Image derived from Z-Stack (Std) of 229 (**c**) or 49 (**d**) images from a gall at 60 dpi completely sectioned in Video S5. (**e**,**f**) Pictures at different magnification of giant cells induced by *Meloidogyne javanica* in cucumber roots at 45–60 dpi. Scale bars: 20 μm. The images were taken from entire galls after fixation with glutaraldehyde and clearing with BABB. Asterisks (*): giant cells. D: dorsal gland; E: egg mass; M: metocorpus; N: nucleus; P: pump chamber.

Galls, GCs, and neighbouring cells at later developmental stages (14, 21, or 60 dpi) could also be fully optically sectioned (Videos S3–S5 for 14, 21, and 60 dpi, respectively). Examples of galls at 14 dpi (Figure 3a,b) and 60 dpi (Figure 3c,d) at different magnifications are shown. At higher magnification (Figure 3a,c), elements of the nematode structures and of the plant interface are observed, as well as subcellular structures of the GCs. For example, at 14 dpi, the dorsal gland and the esophago-intestinal cell are easily distinguished within the nematode (Figure 3a [35]). Additionally, the cytoplasm of the GCs developed their characteristic reticulated form (Figure 3a). At lower magnification, the GCs’ distribution and relative sizes among them could be easily compared (Figure 3b,c). At 60 dpi, an adult female with a developed egg mass can be observed (Figure 3d; Video S5). At these later stages, the observation was made by only placing the sample over a coverslip and into the microscope holder to prevent the flattening of the sample.

The clearing method described here could be also used for the visualization of the GCs induced by *M. javanica* in cucumber roots (Figure 3e–f). Although cucumber roots are thicker than *Arabidopsis* roots, the clearing process allowed the visualization of the GCs formed in cucumber roots even at late stages of infection (45–60 dpi) at higher (Figure 3e) or lower magnification (Figure 3f). 

Hence, we demonstrated that the method described here, taking advantage of the glutaraldehyde auto-fluorescence and the strong clearing effect of the BABB, allowed the sharp visualization of the galls and GCs induced by *M. javanica* in *Arabidopsis* and cucumber roots. This includes a good definition of characteristic GCs’ subcellular structures and a detailed view of the interface between the nematode and the plant, as well as the nematode body at all developmental stages, including at the latest, at life cycle completion, a fully-developed female with a laid egg mass.

### 2.2. 3D Reconstruction of Galls and GCs Induced by M. javanica in Arabidopsis and Cucumber after Optical Sectioning of Fixed and Cleared Galls

Three-dimensional reconstruction of the organs allowed for the measurement of volumes of the GCs or the nuclei inside them. The method proposed here for the clearing-observation of the GCs induced by RKNs allowed us to obtain high-resolution three-dimensional reconstructions, as the voxel width, height, and depth could be adjusted to the limit of the optical characteristics of the microscope and camera. As an example, we obtained 346 optical sections of high-quality captures (voxel-width 0.73 μm/voxel-height 0.73/voxel-depth 0.37 μm) for the three-dimensional reconstruction of an 18 dpi gall in which the nematode and the GCs around its head were clearly visible (Video S6). Other than enabling the visualization of the morphological features of the nematode feeding site, the 346 sections (or as many as desired) obtained with this clearing method could allow, in combination with image processing software, and methods previously described [3,12,14], to obtain volume measurements of the GCs or the subcellular structures as the nuclei. Moreover, in galls at late stages of development, 60 dpi, the GCs can be observed three-dimensionally at full development after dissecting the whole gall with 229 optical sections (Video S7). Similarly, fully-developed GCs from cucumber roots as late as 60 dpi could be 3D reconstructed for the observation of the relationship between the female and the GCs (Video S8).

### 2.3. Assigning a Role for *CTL1* in RKN Feeding Sites by Confocal Based Quantification of GCs’ Size

In order to validate the clearing method for GCs phenotyping (including quantification of GCs’ size), and to increase the knowledge on the plant-RKN interaction, we studied the functional role during the GCs’ formation of a gene that is differentially down-regulated in the GCs’ transcriptome, *CTL1* [22].

We used the plant-nematode transcriptomic tool Nematic [37] to seek biotic stress-related genes specifically down-regulated in the transcriptomes of isolated GCs at early stages of development, as they can be expected to be crucial during the nematode establishment and initial stages of GCs development (Table S1). We filtered those genes fulfilling the following criteria: (i) down-regulated in isolated GCs at 3 dpi (851 genes); (ii) classified as a biotic stress related gene according to Mapman (21 genes); (iii) non-differentially expressed in syncytia induced by *Heterodera schachtii* (in order to select only those genes with expression patterns more specific to the RKN-plant interaction; 12 genes); and (iv) up-regulated at late infection stages, 14 and 21 dpi (to select only those genes actively down-regulated in early stages that presumable could be crucial during GCs formation; 3 genes). After filtering, only three genes (At1g05850, At1g58170, and At3g47540) out of 851 being repressed in GCs fulfilled these four criteria (Table S1). 

**Figure 4:**
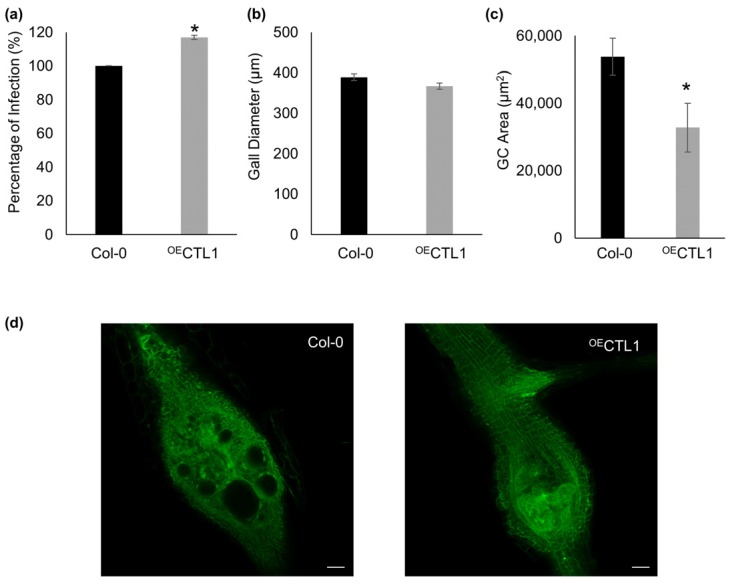
Altered expression of *CTL1* yields a decrease in the GC’s size. (**a**) Infection tests showing the percentage of galls formed in the *CTL1* over-expressing line (^OE^CTL1) compared to Col-0 wild-type. Gall diameter (**b**) and GCs’ size (**c**) at 14 dpi in the control and transgenic line. (**d**) Confocal micrographs of representative galls and GCs at 14 dpi for the different lines. Bars indicate average values of the different parameters measures ± standard errors. Asterisks (*) indicate statistical significance (*p* < 0.05 after students *t*-test; *n* = 5 independent galls, 10 sections each). Scale bars: 50 μm.

We further characterized the role of CTL1 (At1g05850) during GCs’ formation, as it has been suggested to be involved in the cell wall structure [20,38], being well known that the cell wall is altered in nematode-infected roots [6]. A *CTL1* overexpressing line (^OE^CTL1) [20] and a Col-0 wild-type line were inoculated with *M. javanica*. We could observe a significant (*p* < 0.05) increase in the percentage of infection at 14 dpi in the transgenic line compared to the wild-type (*p* < 0.05) although, less than 20% (Figure 4a). Additionally, a clear tendency of reduction of the gall’s diameter in the ^OE^CTL1 line, as compared to Col-0, it was not statistically significant (*p* > 0.05; Figure 4b). In contrast, after measuring the GCs’ area in galls at 14 dpi from the different lines using TrakEM2 [39] from confocal optical sections, the GCs from ^OE^CTL1 lines were significantly smaller (*p* < 0.05; *n* = 5, 10 sections each) than those from the control (Figure 4c,d). All these suggest that a tight regulation of *CTL1* in the GCs is necessary for a proper development of the nematode feeding cells. In this respect, *CTL1* gene encodes a chitinase-related protein with an impact in root architecture in response to mineral cues and possibly with a major role in the control of cell wall structure [20,21,38]. Cell wall modifications are relevant during RKNs’ feeding site development to sustain nematode nourishment because vascular cells from the roots are modified and transformed into transfer cells, like with profuse cell wall ingrowths, forming the GCs [6]. This is probably due to the dramatic reduction of surface area to volume ratio (SA:V) measured after 3D reconstruction along the GCs’ development [3]. The diffusion rate of solutes is proportional to the surface area of the cells. Thus, an increase in the effective solute exchange area is probably achieved through the differentiation of the GCs into transfer cells to compensate for the SA:V reduction as GCs expand. Moreover, *Meloidogyne* spp. and other plant parasitic nematodes secrete cell wall-related proteins during the migratory and establishment phase to loosen the cell walls to establish into the vascular cylinder [6]. Additionally, in response to the nematode presence, several cell wall-related genes, such as expansins, pectinases, or pectate lyases, are up-regulated in the GCs and/or galls [6]. In this respect, the overexpression of a cell wall peroxidase, TPX1, specifically involved in lignification that is repressed in GCs and galls from tomato, caused a severe impairment in the infection and a reduction of GCs’ expansion [23]. All these data highlight the importance of the modifications of the cells wall for proper development and functioning of GCs. Further experiments should be performed to shed light in the putative role of *CTL1* in the modifications of the cell walls in the GCs. 

### 2.4. Clearing and Visualization of Hidden Structures during the Nematode and Other Biotic Interactions

**Figure 5:**
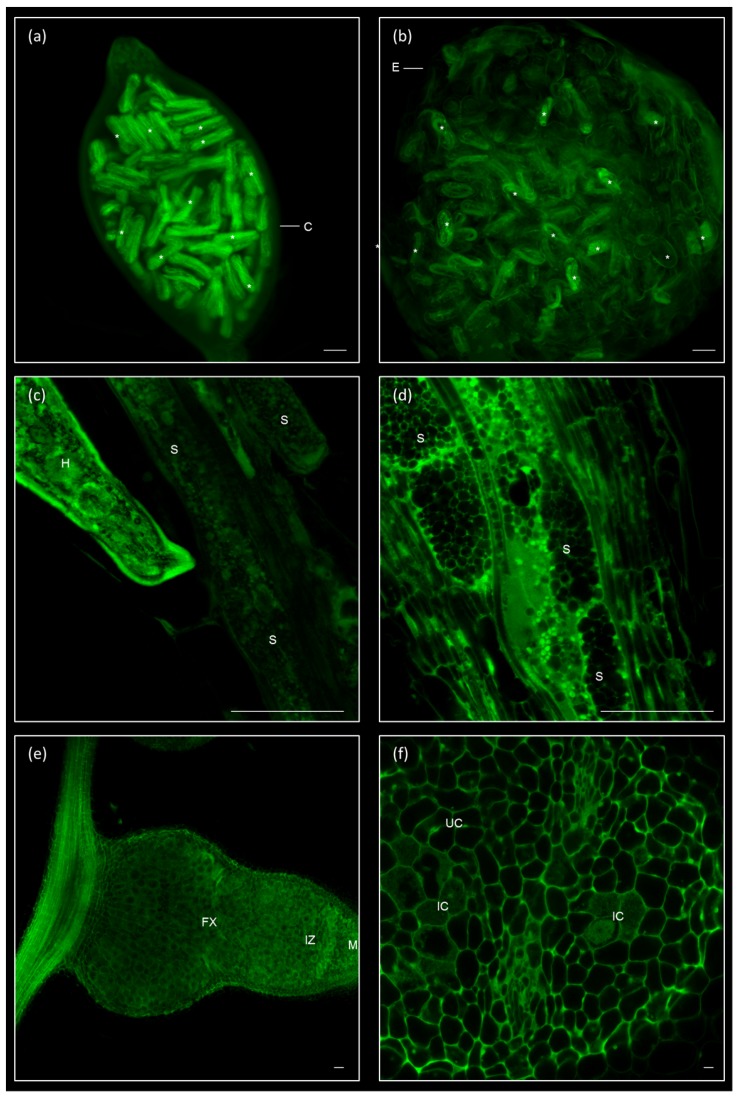
Other biotic interaction can be visualized after fixation and clearing. (**a**) Female of a cyst nematode, *Heterodera schachtii,* filled with eggs one month after infection. (**b**) Egg mass from *Meloidogyne javanica* filled with eggs, two months after infection. (**c**,**d**) Syncytia induced by *Heterodera schachtii* in *Arabidopsis thaliana* at nine days after infection. Details of the nematode protruding the syncytial cells (**c**) or of the dense and multinucleated syncytium (**d**). (**e**,**f**) Nodule induced by *Sinorhizobium meliloti* 2011 in *Medicago truncatula* R108 roots. Longitudinal view with attachment to the root (**e**) or detailed view, or the fixing zone, showing dense infected cells and adjacent uninfected cells (**f**). Scale bars: 50 μm. Asteriks (*): eggs; C: cyst; E: egg mass; H: *Heterodera schachtii*; S: syncytium; M: meristem; IZ: infection zone; FX: fixing zone; IC: infected cell; UC: uninfected cell.

We applied our method to visualize host-parasite structures for which dissection and/or sectioning is usually needed prior to microscopic observation. The cysts are typical resistance structures formed during the reproduction of the plant endoparasitic nematode *Heterodera schachtii*. The female of that species forms a hard and opaque cover to preserve her eggs. After fixing and clearing, the eggs were visualized inside one-month-old cysts without dissection (Figure 5a). Furthermore, we observed the structure and shape of syncytia induced by *H. schachtii* at 9 dpi (Figure 5c,d) and the interface between this cyst nematode and the syncytia, as well as the internal structures of the nematode (Figure 5c) or the dense cytoplasm from the multinucleated syncytial cells (Figure 5d). Root-knot nematodes do not form cysts, but lay a viscous egg mass that protects the eggs inside before hatching. The eggs and juveniles contained inside the egg masses of *M. javanica* at two months post infection could also be clearly observed without dissection (Figure 5b). 

This methodology is also useful to visualize the inner structures of other plant–microbe interactions, such as nodules formed in the rhizobia-legume symbiosis formed by *Sinorhizobium meliloti* and *Medicago truncatula* (Figure 5e,f). Twenty-eight days post invfection nodules were fixed and cleared, and a neat sight of the whole nodule (Figure 5e) could be observed, where we could distinguish the meristem, the infection zone, and the fixing zone. Those are indeterminate nodules that typically present a cylindrical shape, around 1–2 mm thick. A detailed view of the fixing region allowed to clearly differentiate the infected cells, densely occupied by bacteroids, and the uninfected cells (Figure 5f). In Video S9 we show the optical sectioning of a nodule in a transversal view, in which we can track the appearance of two vascular bundles at the poles in sections which go from the meristem, move into the infection zone and, finally, into the fixing zone. 

Therefore, we also demonstrated the utility of this method for the visualization of other plant parasitic nematode structures as the cysts or beneficial biotic interactions as the nodules formed in rhizobia-legume symbiosis. Nodules are specialized organs developed in legume roots to host symbiotic rhizobia, tightly controlled by both symbiotic partners [40,41]. Efforts to clarify the molecular mechanisms governing the nodule formation, have been classically undertaken facing the laborious procedure of embedding, sectioning and staining with toluidine blue to observe tissues inside the nodule [42,43,44,45]. Therefore, the technique presented here would allow for the exploration of several aspects of rhizobia-legume symbiosis, in particular those regarding the first steps in the nodule organogenesis, and in the formation of nodule peripheral tissues.

Additionally, syncytia induced by CNs are easily observed under light microscopy and their occupied area are easily measured [46]. However, the method described in this manuscript could allow not only the visualization of subcellular structures of the syncytial cells, but also that of the eggs inside the cyst. Moreover, the clear visualization of the lumen of the cyst and egg masses could be useful to count the number of eggs, easily distinguishable, as this is one of the reproductive parameters used to assess the infection tests made with these nematodes [46]

In conclusion, we have demonstrated different uses of a fixation and clearing method for the sharp visualization and imaging of galls, in thin (*Arabidopsis*) and thick (cucumber) roots. Importantly, quantification and comparison of feeding cells size was also demonstrated with a wild-type and an overexpression line. In addition, the inner cells and/or tissues of thick dense structures of the plant-nematode interaction and other biotic interactions, such as syncytia, egg masses, cysts, and nodules from *Medicago truncatula* were also clearly distinguishable. Therefore, this method could be of broader utility to other plant-microbe interactions, avoiding the tedious and time consuming embedding and sectioning processes to achieve a proper morphological observation and to phenotype particular structures.

## 3. Materials and Methods

### 3.1. Plant Material, Nematode Population Inoculation, and Infection

*Arabidopsis thaliana* (L.) Heynh Columbia-0 (Col-0) accession was used for all the observation experiments. The *CTL1* overexpressing line (^OE^CTL1, *p35S:CTL1*) was previously described in Hermans et al. [20]. The maintaining of the *Meloidogyne javanica* population in vitro on cucumber roots (*Cucumis sativus* cv. Hoffmanns Giganta) for amplification and egg masses hatching to obtain infective juvenile nematodes was performed according to Díaz-Manzano et al. [47]. Galls obtained from these cucumber plates were also used for the fixation and clearing method described throughout this manuscript. For the inoculation of *Arabidopsis* seedlings with *M. javanica* and the performance of the infection tests, the protocol described in Olmo et al. [19] was followed. Three independent infection tests with at least 180 plants per line were performed and the number of galls was scored at 14 days post infection (dpi). The *Heterodera schachtii* population maintenance in mustard seedlings (*Sinapis alba* cv. Albatros), the stimulation of egg hatching in sterile 3 mM ZnCl_2_ and the infection were performed accordingly to Bohlmann and Wieczorek [46]. 

*Medicago truncatula* R108 seeds were scarified by incubation for 7 min with concentrated H_2_SO_4_, washed with water, surface sterilized with 50% bleach for 90 s and left overnight in sterile water. After 48 h at 4 °C, seeds were germinated in water-agar plates at 22 °C for 24 h, then transplanted to Fahreus [48] agar plates (1% agar) without nitrogen, and inoculated with *S. meliloti* 2011. Plants were cultivated vertically in a phytochamber set to 16 h of light and 22 °C conditions. Nodulation was checked weekly under a stereomicroscope. After 10 dpi several nodule primordia were observed. Finally, mature nitrogen-fixing nodules, presenting their typical red colour appearance, were collected at 28 dpi and fixed and cleared as mentioned below.

### 3.2. Fixation and Clearing of the Biological Samples

Galls, syncytia, nodules, cysts, and egg masses were hand-dissected and collected immediately in 50 mM sodium-phosphate buffer (pH 7) at room temperature. The samples were fixed in the same buffer with 3% (*v*/*v*) glutaraldehyde (around 10 galls/syncytia/females per mL or 5 nodules per mL) under vacuum for 15 min and maintained overnight (or prolonged time period) at 4 °C. After fixation, the samples were rinsed twice for 5 min with sodium phosphate buffer to eliminate glutaraldehyde residues. The samples were sequentially dehydrated for 20 min in solutions of 30%, 50%, 70%, and 90% (*v*/*v*) ethanol (all dilutions in phosphate-buffered saline, pH 7), and kept overnight in pure ethanol (around 10 galls/syncytia/females per mL or 5 nodules per mL). 

For the subsequent clearing process, the samples were transferred to a solution 1:1 *v*/*v* of EtOH: BABB (BABB: benzyl alcohol (Sigma 402834; St. Louis, MO, USA)/benzyl benzoate (Sigma B6630; St. Louis, MO, USA) mixed in a 1:2 proportion (*v*/*v*) for 20 min at room temperature (around 10 galls/syncytia/females per mL or 5 nodules per mL). Lastly, the samples were maintained at least 20 min in 100% BABB at room temperature before observation under the confocal microscope. Twenty minutes was enough for root material, however, for thicker or harder structures, it is convenient to maintain the samples at least overnight. At this step, the samples could be storage at 4 °C in 100% BABB for longer time periods. 

### 3.3. Confocal Microscopy

Cleared samples were observed with a Leica TCS SP2 laser scanning confocal microscope (Leica, Wetzlar, Germany). Glutaraldehyde auto-fluorescence was excited with a 488 nm Ar/Kr laser. The green auto-fluorescence generated by the glutaraldehyde was recovered for image capture in the range of 500–600 nm. Excitation Beam Splitter FW RSP 500 was activated. The pinhole was adjusted to 1 Airy unit. Scan speed was settled to 400 Hz. For three-dimensional reconstruction, samples were directly observed on a coverslip placed in the microscope holder, so that the sample would not be flattened. Leica Confocal Software LCS was used for capturing the images. 

### 3.4. Image Processing

ImageJ basic package [49] was used to analyse the image stacks and to obtain movies, montages, Z-projections, and three-dimensional reconstructions. All the options can be found under the “Image-Stacks” menu in the ImageJ tool bar. Gall diameters were measured by using the straightforward line tool from the same ImageJ basic package. Giant cell areas were measured according to Cabrera et al. [3]. Briefly, the average area occupied by all the GCs (normally 5–8) in the ten sections where they showed a maximum enlargement, was measured from five independent galls per line by using the ImageJ (https://imagej.nih.gov/ij/) plugin TrakEM2 [3,39]. The average size area and standard error for each value were calculated. For statistical analysis, a Student’s *t*-test was performed (*p* < 0.05).

## Supplementary Materials

Supplementary materials can be found at http://www.mdpi.com/1422-0067/19/2/429/s1 and www.mdpi.com/1422-0067/19/2/429/s2.
