# Supplementary material for: A Phenotyping Method of Giant Cells from Root-Knot Nematode Feeding Sites by Confocal Microscopy Highlights a Role for CHITINASE-LIKE 1 in Arabidopsis
Source: Int J Mol Sci. 2018 Jan 31;19(2):429. doi: 10.3390/ijms19020429 (PMC5855651; doi:10.3390/ijms19020429)
Supplement: Supplementary file 1 [file ijms-19-00429-s001.zip › Supplementary-1/Table S1.pdf]

International Journal of
Molecular Sciences

MDPI

Article

A Phenotyping Method of Giant Cells from Root-Knot Nematode Feeding Sites by Confocal Microscopy Highlights a Role for *CHITINASE-LIKE 1* in *Arabidopsis*

Javier Cabrera [1], Rocio Olmo [1], Virginia Ruiz-Ferrer [1], Isidro Abreu [2], Christian Hermans [3], Isabel Martinez-Argudo [4], Carmen Fenoll [1] and Carolina Escobar [1,*]

Table S1. List of genes differentially expressed in nematode feeding sites selected by the following criteria: Down- regulated in isolated GCs at 3 dpi and classified as a biotic stress related gene according to Mapman (21 genes), non-differentially expressed in syncytia induced by *Heterodera schachtii* (12 genes), and up- regulated at older stages of infection, 14 and 21 dpi (3 genes in bold).

AGI code	Description	GC 3dpi	Gall 3dpi	Gall 7dpi	Gall 14dpi	Gall 21dpi	Syncytia 5+15dpi
**At1g05850**	**POM1: Encodes an endo chitinase-like protein AtCTL1. Essential for tolerance to heat, salt and drought stresses. Also involved in root hair development, cell expansion and response to cytokinin.**	**−1.48**	**NDE**	**NDE**	**1.12**	**0.89**	**NDE**
**At1g58170**	**disease resistance-responsive protein-related / dirigent protein-related**	**−1.55**	**NDE**	**NDE**	**1.41**	**0.70**	**NDE**
At1g65390	PP2-A5: Symbols: ATPP2-A5 | ATPP2-A5 (ARABIDOPSIS THALIANA PHLOEM PROTEIN 2 A5); carbohydrate binding	−1.39	NDE	NDE	NDE	−0.70	NDE
At1g73260	KTI1: Encodes a trypsin inhibitor involved in modulating programmed cell death in plant-pathogen interactions.	−3.05	NDE	NDE	−2.09	−2.01	−5.80
At1g75030	TLP-3: encodes a PR5-like protein	−1.17	NDE	NDE	NDE	NDE	NDE
At2g14610	PR1: PR1 gene expression is induced in response to a variety of pathogens. Expression of this gene is salicylic-acid responsive.	−4.07	NDE	NDE	NDE	NDE	NDE
At2g21100	disease resistance-responsive protein-related / dirigent protein-related	−1.13	NDE	NDE	NDE	NDE	−5.10

International Journal of *Molecular Sciences*

MDPI

At3g04720	PR4: Encodes a protein similar to the antifungal chitin-binding protein hevein from rubber tree latex. mRNA levels increase in response to ethylene and turnip crinkle virus infection.	−4.15	NDE	NDE	−1.91	−1.15	−5.60
At3g17210	HS1: Encodes a heat stable protein with antimicrobial and antifungal activity.	−1.21	NDE	NDE	NDE	NDE	1.30
At3g28940	avirulence-responsive protein, putative / avirulence induced gene (AIG) protein, putative	−1.92	NDE	NDE	NDE	NDE	NDE
At3g44670	ATP binding / nucleoside-triphosphatase/ nucleotide binding / protein binding / transmembrane receptor	−1.32	NDE	NDE	NDE	NDE	NDE
**At3g47540**	**chitinase, putative**	**−1.40**	**−1.02**	**0.69**	**1.21**	**1.07**	**NDE**
At3g50460	HR2: Homolog of RPW8	−0.97	NDE	NDE	NDE	NDE	NDE
At3g59930	Encodes a defensin-like (DEFL) family protein.	−4.03	NDE	NDE	NDE	NDE	NDE
At4g09940	avirulence-responsive family protein / avirulence induced gene (AIG1) family protein	−1.43	NDE	NDE	NDE	NDE	NDE
At4g33720	pathogenesis-related protein, putative	−0.80	NDE	NDE	NDE	NDE	−5.40
At5g39730	avirulence-responsive protein-related / avirulence induced gene (AIG) protein-related	−1.81	NDE	NDE	NDE	NDE	2.70
At5g40910	disease resistance protein (TIR-NBS-LRR class), putative	−1.18	NDE	NDE	NDE	NDE	−2.00
At5g45050	TTR1: Encodes a member of WRKY Transcription Factor (Group II-e) that confers resistance to tobacco ringspot nepovirus.	−1.59	NDE	NDE	NDE	NDE	NDE
At5g51630	disease resistance protein (TIR-NBS-LRR class), putative	−1.25	NDE	NDE	NDE	NDE	−2.50
At5g64930	CPR5: Regulator of expression of pathogenesis-related (PR) genes.	−0.81	NDE	NDE	0.89	0.78	−2.00
